# A Comparison of Shell Theories for Vibration Analysis of Single-Walled Carbon Nanotubes Based on an Anisotropic Elastic Shell Model

**DOI:** 10.3390/nano13081390

**Published:** 2023-04-17

**Authors:** Matteo Strozzi, Isaac E. Elishakoff, Michele Bochicchio, Marco Cocconcelli, Riccardo Rubini, Enrico Radi

**Affiliations:** 1 Department of Sciences and Methods for Engineering, University of Modena and Reggio Emilia, 42122 Reggio Emilia, Italy; matteo.strozzi@unimore.it (M.S.); marco.cocconcelli@unimore.it (M.C.); riccardo.rubini@unimore.it (R.R.); enrico.radi@unimore.it (E.R.); 2 Department of Ocean and Mechanical Engineering, Florida Atlantic University, Boca Raton, FL 33431, USA

**Keywords:** carbon nanotubes, vibration analysis, anisotropic elastic model, shell theories, natural frequencies

## Abstract

In the present paper, a comparison is conducted between three classical shell theories as applied to the linear vibrations of single-walled carbon nanotubes (SWCNTs); specifically, the evaluation of the natural frequencies is conducted via Donnell, Sanders, and Flügge shell theories. The actual discrete SWCNT is modelled by means of a continuous homogeneous cylindrical shell considering equivalent thickness and surface density. In order to take into account the intrinsic chirality of carbon nanotubes (CNTs), a molecular based anisotropic elastic shell model is considered. Simply supported boundary conditions are imposed and a complex method is applied to solve the equations of motion and to obtain the natural frequencies. Comparisons with the results of molecular dynamics simulations available in literature are performed to check the accuracy of the three different shell theories, where the Flügge shell theory is found to be the most accurate. Then, a parametric analysis evaluating the effect of diameter, aspect ratio, and number of waves along the longitudinal and circumferential directions on the natural frequencies of SWCNTs is performed in the framework of the three different shell theories. Assuming the results of the Flügge shell theory as reference, it is obtained that the Donnell shell theory is not accurate for relatively low longitudinal and circumferential wavenumbers, for relatively low diameters, and for relatively high aspect ratios. On the other hand, it is found that the Sanders shell theory is very accurate for all the considered geometries and wavenumbers, and therefore, it can be correctly adopted instead of the more complex Flügge shell theory for the vibration modelling of SWCNTs.

## 1. Introduction

Since their discovery in 1991 in Japan in the laboratories of the NEC Corporation by Professor Sumio Iijima [1], the study of the vibrations of carbon nanotubes has represented a very demanding challenge for many researchers all over the world.

This interest in carbon nanotubes is due to their extraordinary mechanical properties, in particular the very high elastic modulus and tensile strength, together with their very small diameter, which allows them to reach natural frequencies of the THz order, and therefore leads them to be applied in several high sensitivity electro-mechanical systems, such as resonators, sensors, and oscillators [2,3,4,5,6].

In order to study the vibratory behaviour of carbon nanotubes, three different methods have been proposed: experimental analyses, molecular dynamics simulations, and continuous models.

The experimental analyses, conducted on the basis of resonant Raman spectroscopy, allow to obtain the natural frequencies only of peculiar modes of carbon nanotubes, the so-called “radial breathing modes”, which are characterized by zero waves both longitudinal and circumferential, as undeformed axisymmetric modes [7,8,9]. In addition to this, the main limitation of the experimental analyses is due to their very high technical difficulty and the need to work with scanning or transmission electron microscopes with very high resolution (and therefore very high cost).

Molecular dynamics simulations take into account the discrete nature of carbon nanotubes by modelling bonds and interaction forces between the different carbon atoms based on the fundamental concepts of molecular mechanics [10]. These analyses allow to obtain the natural frequencies of both radial breathing and beam-like modes, where the latter are very important for carbon nanotubes, which have high aspect ratio (i.e., the ratio between length and radius) and, therefore, have a vibratory behaviour very similar to beam structures [11,12]. The main limitation of molecular dynamics simulations results from the high computational effort needed to carry out numerical analyses, especially in the presence of a large number of carbon atoms, a condition that usually occurs in carbon nanotubes, due to the fact that they are long and of reduced thickness (i.e., the ratio between thickness and radius), which leads them to be considered “thin-walled structures”.

As an alternative to the experimental analyses and numerical simulations, researchers have proposed several continuous models, mainly of beam-type or shell-type, to study carbon nanotube vibrations via continuum mechanics [13].

In particular, the continuous beam-type models are able to properly simulate the torsional vibrations of nanotubes [14], whereas the continuous shell-type models are able to also properly simulate the flexural vibrations of nanotubes, which give the highest natural frequencies; they therefore represent more complete models [15].

In general, the main problem in the continuous modelling of carbon nanotubes is the choice of the equivalent parameters that allow to study the discrete nanotubes as continuous structures.

For the shell models, which as previously mentioned are the most accurate ones, Yakobson [16], starting from results of molecular dynamics simulations, proposed equivalent values of tensile and flexural stiffness, and therefore thickness and surface density, thus allowing the study of carbon nanotubes as continuous isotropic cylindrical shells.

Considering an isotropic elastic shell model and using these equivalent parameters, several papers have been published concerning the study of the natural frequencies of single- or multi-walled carbon nanotubes in the framework of thin shell theories; these principally include Donnell-Mushtari, Sanders-Koiter, and Lur’ye-Flügge-Byrne [17,18,19,20].

Readers interested in deepening the peculiarities of these thin shell theories can find the related strain–displacement relationships and equations of motion in the fundamental monographs by Leissa [21], Yamaki [22], Amabili [23], Soedel [24], and Ventsel [25].

An interesting comparison of thin shell theories for vibrations of circular cylindrical shells was carried out by Amabili [26]. It was derived from this work that, among the Donnell, Flügge, and Sanders shell theories, the Donnell shell theory, which has the lowest analytical complexity (i.e., the lowest number of terms in the equations of motion), presents the lowest accuracy in modelling shell vibrations. On the other hand, it was found that the Flügge shell theory, which has the highest analytical complexity, presents the highest accuracy. Finally, the Sanders shell theory, which has an intermediate analytical complexity, presents a relatively high accuracy. Moreover, an examination of cylindrical shell theories, specifically Donnell and Sanders shell theories, for the buckling of carbon nanotubes was performed by Wang et al. [27].

The same continuous models adopted to predict CNT vibrations at the nanoscopic scale can be used at the macroscopic scale to investigate the vibrations of different configurations of circular cylindrical shells, e.g., FGM shells [28,29,30], multi-layer composite shells [31,32], sandwich composite shells [33], and laminated orthotropic shells [34], which are considered in several mechanical components. It is important to stress that, in the passage from the nanoscopic to macroscopic scale, the continuous models lose the anisotropic behaviour due to the inherent chirality of carbon nanotubes.

However, given the intrinsically anisotropic nature of carbon nanotubes, in order to correctly study their vibratory behaviour and also to take into account the dependence of their elastic properties on chirality, it is necessary to adopt an anisotropic model.

To this aim, Chang [35,36], starting from considerations of molecular mechanics, developed a novel and very accurate anisotropic elastic shell model capable of correctly predicting the dependence of the material elastic properties on CNT chirality and dimensions, and therefore, also able to calculate the natural frequencies very similar to those obtained via molecular dynamics simulations.

Adopting this anisotropic elastic model, the linear vibrations of single-walled and multi-walled carbon nanotubes for different geometries and wave numbers were investigated separately via the Donnell [37], Sanders [38], and Flügge [39,40] thin shell theories.

To the knowledge of the authors of this paper, a study on the linear vibrations of SWCNTs based on an anisotropic elastic shell model that compares the values obtained for the natural frequencies by applying the Donnell, Sanders, and Flügge thin shell theories for different geometries and wavenumbers has not yet been published in the literature. In fact, the authors of this paper believe that it could be very useful to investigate the field of applicability and limitation of the three previously indicated thin shell theories in order to identify which of them is able to provide sufficiently accurate results with a relatively low computational effort.

To this aim, in the present paper, the natural frequencies of SWCNTs are obtained in the framework of the Donnell, Sanders, and Flügge shell theories, where the actual discrete SWCNT is modelled via a continuous homogeneous cylindrical shell considering equivalent thickness and surface density. An anisotropic elastic shell model is adopted to take into account the intrinsic chirality effects of CNTs. Simply supported boundary conditions are imposed and the complex method is used to solve the dynamic equations of motion and to obtain the natural frequencies. Vibration modes with different numbers of longitudinal and circumferential waves are studied. SWCNTs with different geometries are analysed.

Taking the more accurate but more complex Flügge theory as a reference, the main objective of this work was to establish whether the simpler Donnell or Sanders shell theories allow to obtain sufficiently accurate natural frequencies and, therefore, can be adopted instead of the Flügge theory to correctly model the linear vibrations of SWCNTS on the basis of an anisotropic elastic shell model.

## 2. Thin Shell Theories for SWCNTs

**Figure 1:**
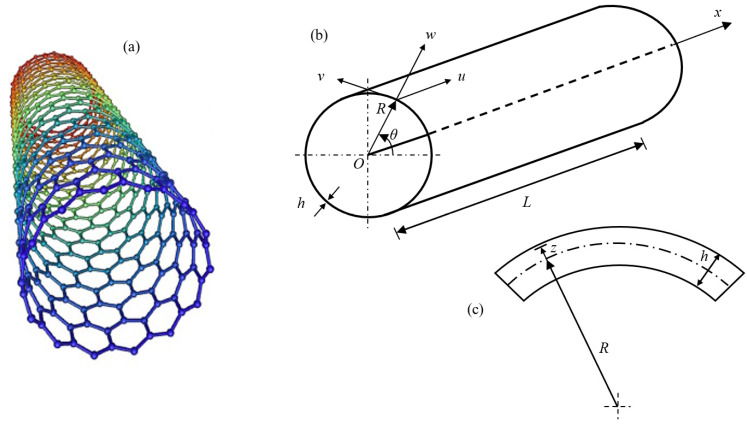
Continuous modelling of SWCNTs: (**a**) actual discrete SWCNT; (**b**) geometry of the equivalent continuous circular cylindrical shell; and (**c**) cross-section of the surface of the equivalent continuous circular cylindrical shell [20].

In the present paper, the actual discrete SWCNT of Figure 1a is modelled by means of an equivalent continuous elastic thin cylindrical shell, see Figure 1b,c with radius R, length L, and thickness h. A cylindrical coordinate system (O,x,θ,z) is adopted, where the origin O of the reference system is located at the centre of one end of the cylindrical shell. Three displacements are present: longitudinal u(x,θ,t), circumferential v(x,θ,t), and radial w(x,θ,t), where the radial displacement w is assumed as positive outward, (x,θ) are the longitudinal and angular coordinates of an arbitrary point on the middle surface of the shell, z is the radial coordinate along the thickness h, and t is the time.

In this paper, the natural frequencies obtained by considering three different thin shell theories are compared regarding the linear vibrations of SWCNTs with different geometries and wavenumbers. These theories are based on Love’s first approximation assumptions [21]: (i) the thickness h of the shell is small with respect to the radius of curvature R of the middle surface; (ii) the strains are small; (iii) the transverse normal stress is small; (iv) and the normal to the undeformed middle surface remains straight and normal to the middle surface after the deformation, and undergoes no thickness stretching (Kirchhoff-Love kinematic hypothesis). The considered thin shell theories are: (a) Donnell-Mushtari [23], (b) Sanders-Koiter [24], and (c) Flügge-Lur’ye-Byrne [25]; for all of them, both rotary inertia and shear deformations are neglected.

### 2.1. Strain–Displacement Relationships

According to the Donnell, Sanders, and Flügge shell theories, the middle surface strains $\left(\varepsilon_{x,0},\varepsilon_{\theta ,0},\gamma_{x\theta ,0}\right)$ of the shell are related to the displacements (u,v,w) in the form [21]:

(1) $$\varepsilon_{x,0}=\frac{\partial u}{\partial x}\;\varepsilon_{\theta ,0}=\frac{1}{R}\frac{\partial v}{\partial \theta }+\frac{w}{R}\;\gamma_{x\theta ,0}=\frac{\partial v}{\partial x}+\frac{1}{R}\frac{\partial u}{\partial \theta }$$

From Equation (1), it can be observed that the middle surface strains are expressed in the same form for the three different shell theories considered.

According to Donnell, Sanders, and Flügge, the middle surface changes in the curvature and torsion $\left(k_{x},k_{\theta },k_{x\theta }\right)$ of the shell are related to the displacements (u,v,w) in the form [21]:

(2) $$\begin{array}{c} k_{x}=-\frac{\partial^{2}w}{\partial x^{2}}\;k_{\theta }=\frac{1}{R^{2}}\left(-\frac{\partial^{2}w}{\partial \theta^{2}}+\psi \frac{\partial v}{\partial \theta }-\varphi w\right)\\ k_{x\theta }=-\frac{2}{R}\frac{\partial^{2}w}{\partial x\partial \theta }-\frac{1}{R^{2}}\left(\frac{1}{2}\psi +\varphi \right)\frac{\partial u}{\partial \theta }+\frac{1}{R}\left(\frac{3}{2}\psi +\varphi \right)\frac{\partial v}{\partial x} \end{array}$$

From Equation (2), it can be noted that the middle surface change in the curvature $k_{\theta }$ and torsion $k_{x\theta }$ are written in a different form for the three different shell theories, where parameters (ψ=0,φ=0) denote the Donnell shell theory, parameters (ψ=1,φ=0) denote the Sanders shell theory, and parameters (ψ=0,φ=1) denote the Flügge shell theory. Since the Sanders and Flügge shell theories have more terms in the Expansion (2) than the Donnell shell theory, the first two theories can be expected to be more accurate than the third in the modelling of SWCNT linear vibrations.

### 2.2. Strain Components at an Arbitrary Point of the Shell Surface

According to the Donnell, Sanders and Flügge shell theories, the strain components $\left(\varepsilon_{x},\varepsilon_{\theta },\gamma_{x\theta }\right)$ at an arbitrary point of the surface of the shell are related to the middle surface strains $\left(\varepsilon_{x,0},\varepsilon_{\theta ,0},\gamma_{x\theta ,0}\right)$ and to the changes in curvature and torsion of the middle surface $\left(k_{x},k_{\theta },k_{x\theta }\right)$ by the relationships [23]:

(3) $$\varepsilon_{x}=\varepsilon_{x,0}+zk_{x}\;\varepsilon_{\theta }=\varepsilon_{\theta ,0}+zk_{\theta }\;\gamma_{x\theta }=\gamma_{x\theta ,0}+zk_{x\theta }$$

where z is the distance of the considered arbitrary point of the shell from the middle surface.

Substituting Equations (1) and (2) into Equation (3), the following is obtained:

(4) $$\begin{array}{c} \varepsilon_{x}=\frac{\partial u}{\partial x}-z\frac{\partial^{2}w}{\partial x^{2}}\;\varepsilon_{\theta }=\frac{1}{R}\frac{\partial v}{\partial \theta }+\frac{w}{R}+\frac{z}{R^{2}}\left(-\frac{\partial^{2}w}{\partial \theta^{2}}+\psi \frac{\partial v}{\partial \theta }-\varphi w\right)\\ \gamma_{x\theta }=\frac{\partial v}{\partial x}+\frac{1}{R}\frac{\partial u}{\partial \theta }-\frac{2z}{R}\frac{\partial^{2}w}{\partial x\partial \theta }-\frac{z}{R^{2}}\left(\frac{1}{2}\psi +\varphi \right)\frac{\partial u}{\partial \theta }+\frac{z}{R}\left(\frac{3}{2}\psi +\varphi \right)\frac{\partial v}{\partial x} \end{array}$$

where Equation (4) relate the strain components at an arbitrary point of the shell surface $\left(\varepsilon_{x},\varepsilon_{\theta },\gamma_{x\theta }\right)$ to the displacements (u,v,w).

## 3. Anisotropic Elastic Shell Model

Considering the molecular based anisotropic elastic shell model developed by Chang [35,36], which includes the chirality effects characteristic of SWCNTs, the stress–strain relationships can be written as:

(5) $$\begin{array}{c} \sigma_{x}=\frac{1}{h}\left(Y_{11}\varepsilon_{x}+Y_{12}\varepsilon_{\theta }+Y_{13}\gamma_{x\theta }\right)\\ \sigma_{\theta }=\frac{1}{h}\left(Y_{21}\varepsilon_{x}+Y_{22}\varepsilon_{\theta }+Y_{23}\gamma_{x\theta }\right)\\ \tau_{x\theta }=\frac{1}{h}\left(Y_{31}\varepsilon_{x}+Y_{32}\varepsilon_{\theta }+Y_{33}\gamma_{x\theta }\right) \end{array}$$

where $\left(\sigma_{x},\sigma_{\theta },\tau_{x\theta }\right)$ are the stress components at an arbitrary point of the shell surface, and $Y_{ij}$ are the anisotropic surface elastic constants of an arbitrary SWCNT, which are defined as [35,36]:

(6) $$Y_{ij}=\frac{2}{3\sqrt{3}}\left(K_{\rho }G_{li}G_{lj}+2\frac{K_{\theta }}{a^{2}}H_{li}H_{lj}\right),\;i,j,l=1,2,3(\mathrm{sum over}\;l)$$

in which a is the carbon–carbon bond length; $\left(K_{\rho },K_{\theta }\right)$ are force constants associated with stretching and angular distortion of the carbon-carbon bond, respectively, where these constants can be obtained from quantum (ab initio) mechanics, empirical molecular potential, or fitted to experimental data; and $\left(G_{li},G_{lj},H_{li},H_{lj}\right)$ are elements of matrices G and H, respectively, which are given in detail in Ref. [36].

(7) $$\begin{array}{cc} \sigma_{x} & =\frac{1}{h}\left\{Y_{11}\left(\frac{\partial u}{\partial x}-z\frac{\partial^{2}w}{\partial x^{2}}\right)+Y_{12}\left[\frac{1}{R}\frac{\partial v}{\partial \theta }+\frac{w}{R}+\frac{z}{R^{2}}\left(-\frac{\partial^{2}w}{{\partial \theta }^{2}}+\psi \frac{\partial v}{\partial \theta }-\varphi w\right)\right]\right.\\ & \left.+Y_{13}\left[\frac{\partial v}{\partial x}+\frac{1}{R}\frac{\partial u}{\partial \theta }-\frac{2z}{R}\frac{\partial^{2}w}{\partial x\partial \theta }-\frac{z}{R^{2}}\left(\frac{1}{2}\psi +\varphi \right)\frac{\partial u}{\partial \theta }+\frac{z}{R}\left(\frac{3}{2}\psi +\varphi \right)\frac{\partial v}{\partial x}\right]\right\} \end{array}$$

(8) $$\begin{array}{cc} \sigma_{\theta } & =\frac{1}{h}\left\{Y_{21}\left(\frac{\partial u}{\partial x}-z\frac{\partial^{2}w}{\partial x^{2}}\right)+Y_{22}\left[\frac{1}{R}\frac{\partial v}{\partial \theta }+\frac{w}{R}+\frac{z}{R^{2}}\left(-\frac{\partial^{2}w}{{\partial \theta }^{2}}+\psi \frac{\partial v}{\partial \theta }-\varphi w\right)\right]\right.\\ & \left.+Y_{23}\left[\frac{\partial v}{\partial x}+\frac{1}{R}\frac{\partial u}{\partial \theta }-\frac{2z}{R}\frac{\partial^{2}w}{\partial x\partial \theta }-\frac{z}{R^{2}}\left(\frac{1}{2}\psi +\varphi \right)\frac{\partial u}{\partial \theta }+\frac{z}{R}\left(\frac{3}{2}\psi +\varphi \right)\frac{\partial v}{\partial x}\right]\right\} \end{array}$$

(9) $$\begin{array}{cc} \tau_{x\theta } & =\frac{1}{h}\left\{Y_{31}\left(\frac{\partial u}{\partial x}-z\frac{\partial^{2}w}{\partial x^{2}}\right)+Y_{32}\left[\frac{1}{R}\frac{\partial v}{\partial \theta }+\frac{w}{R}+\frac{z}{R^{2}}\left(-\frac{\partial^{2}w}{{\partial \theta }^{2}}+\psi \frac{\partial v}{\partial \theta }-\varphi w\right)\right]\right.\\ & \left.+Y_{33}\left[\frac{\partial v}{\partial x}+\frac{1}{R}\frac{\partial u}{\partial \theta }-\frac{2z}{R}\frac{\partial^{2}w}{\partial x\partial \theta }-\frac{z}{R^{2}}\left(\frac{1}{2}\psi +\varphi \right)\frac{\partial u}{\partial \theta }+\frac{z}{R}\left(\frac{3}{2}\psi +\varphi \right)\frac{\partial v}{\partial x}\right]\right\} \end{array}$$

## 4. Equations of Motion

The general equations of motion for an arbitrary SWCNT in terms of force $\left(N_{x},N_{\theta },N_{x\theta }\right)$ and moment $\left(M_{x},M_{\theta },M_{x\theta }\right)$ resultants are written in the form [22]:

(10) $$\begin{array}{c} \frac{\partial N_{x}}{\partial x}+\frac{1}{R}\frac{\partial N_{x\theta }}{\partial \theta }-\frac{1}{2R^{2}}\frac{\partial M_{x\theta }}{\partial \theta }-\rho h\frac{\partial^{2}u}{{\partial t}^{2}}=0\\ \frac{1}{R}\frac{\partial N_{\theta }}{\partial \theta }+\frac{\partial N_{x\theta }}{\partial x}+\frac{3}{2R}\frac{\partial M_{x\theta }}{\partial x}+\frac{1}{R^{2}}\frac{\partial M_{\theta }}{\partial \theta }-\rho h\frac{\partial^{2}v}{{\partial t}^{2}}=0\\ \frac{\partial^{2}M_{x}}{{\partial x}^{2}}+\frac{2}{R}\frac{\partial^{2}M_{x\theta }}{\partial x\partial \theta }+\frac{1}{R^{2}}\frac{\partial^{2}M_{\theta }}{{\partial \theta }^{2}}-\frac{N_{\theta }}{R}-\rho h\frac{\partial^{2}w}{{\partial t}^{2}}=0 \end{array}$$

where ρh is the mass density per unit lateral area (i.e., the surface density) of SWCNT.

In the anisotropic elastic shell model, the force and moment resultants are defined based on the stress components in Equation (5), in the form [39]:

(11) $$\begin{array}{c} N_{x}=\int_{-h/2}^{h/2}\sigma_{x}\left(1+\varphi \frac{z}{R}\right)dz=Y_{11}\frac{\partial u}{\partial x}+\frac{Y_{12}}{R}\left(\frac{\partial v}{\partial \theta }+w\right)+Y_{13}\left(\frac{\partial v}{\partial x}+\frac{1}{R}\frac{\partial u}{\partial \theta }\right)\\ +\varphi \left[-\frac{X_{11}}{R}\frac{\partial^{2}w}{{\partial x}^{2}}\right.\left.-\frac{X_{12}}{R}\left(\frac{1}{R^{2}}\frac{\partial^{2}w}{{\partial \theta }^{2}}\right.\left.+\frac{w}{R^{2}}\right)+\frac{X_{13}}{R}\left(-\frac{2}{R}\frac{\partial^{2}w}{\partial x\partial \theta }-\frac{1}{R^{2}}\frac{\partial u}{\partial \theta }+\frac{1}{R}\frac{\partial v}{\partial x}\right)\right] \end{array}$$

(12) $$N_{\theta }=\int_{-h/2}^{h/2}\sigma_{\theta }dz=Y_{21}\frac{\partial u}{\partial x}+\frac{Y_{22}}{R}\left(\frac{\partial v}{\partial \theta }+w\right)+Y_{23}\left(\frac{\partial v}{\partial x}+\frac{1}{R}\frac{\partial u}{\partial \theta }\right)$$

(13) $$\begin{array}{c} N_{x\theta }=\int_{-h/2}^{h/2}\tau_{x\theta }\left(1+\varphi \frac{z}{R}\right)dz=Y_{31}\frac{\partial u}{\partial x}+\frac{Y_{32}}{R}\left(\frac{\partial v}{\partial \theta }+w\right)+Y_{33}\left(\frac{\partial v}{\partial x}+\frac{1}{R}\frac{\partial u}{\partial \theta }\right)\\ +\varphi \left[-\frac{X_{31}}{R}\frac{\partial^{2}w}{{\partial x}^{2}}\right.\left.-\frac{X_{32}}{R}\left(\frac{1}{R^{2}}\frac{\partial^{2}w}{{\partial \theta }^{2}}\right.\left.+\frac{w}{R^{2}}\right)+\frac{X_{33}}{R}\left(-\frac{2}{R}\frac{\partial^{2}w}{\partial x\partial \theta }-\frac{1}{R^{2}}\frac{\partial u}{\partial \theta }+\frac{1}{R}\frac{\partial v}{\partial x}\right)\right] \end{array}$$

(14) $$\begin{array}{c} M_{x}=\int_{-h/2}^{h/2}\sigma_{x}\left(1+\varphi \frac{z}{R}\right)zdz=-X_{11}\frac{\partial^{2}w}{{\partial x}^{2}}+\frac{X_{12}}{R^{2}}\left(-\frac{\partial^{2}w}{{\partial \theta }^{2}}+\psi \frac{\partial v}{\partial \theta }-\varphi w\right)\\ +X_{13}\left[-\frac{2}{R}\frac{\partial^{2}w}{\partial x\partial \theta }\right.-\frac{1}{R^{2}}\left(\frac{1}{2}\psi +\varphi \right)\left.\frac{\partial u}{\partial \theta }+\frac{1}{R}\left(\frac{3}{2}\psi +\varphi \right)\frac{\partial v}{\partial x}\right]\\ +\varphi \left[\frac{X_{11}}{R}\frac{\partial u}{\partial x}+\frac{X_{12}}{R^{2}}\left(\frac{\partial v}{\partial \theta }+w\right)+\frac{X_{13}}{R}\left(\frac{\partial v}{\partial x}+\frac{1}{R}\frac{\partial u}{\partial \theta }\right)\right] \end{array}$$

(15) $$\begin{array}{c} M_{\theta }=\int_{-h/2}^{h/2}\sigma_{\theta }zdz=-X_{21}\frac{\partial^{2}w}{{\partial x}^{2}}+\frac{X_{22}}{R^{2}}\left(-\frac{\partial^{2}w}{{\partial \theta }^{2}}+\psi \frac{\partial v}{\partial \theta }-\varphi w\right)\\ +X_{23}\left[-\frac{2}{R}\frac{\partial^{2}w}{\partial x\partial \theta }-\frac{1}{R^{2}}\left(\frac{1}{2}\psi +\varphi \right)\frac{\partial u}{\partial \theta }+\frac{1}{R}\left(\frac{3}{2}\psi +\varphi \right)\frac{\partial v}{\partial x}\right] \end{array}$$

(16) $$\begin{array}{c} M_{x\theta }=\int_{-h/2}^{h/2}\tau_{x\theta }\left(1+\varphi \frac{z}{R}\right)zdz=-X_{31}\frac{\partial^{2}w}{{\partial x}^{2}}+\frac{X_{32}}{R^{2}}\left(-\frac{\partial^{2}w}{{\partial \theta }^{2}}+\psi \frac{\partial v}{\partial \theta }-\varphi w\right)\\ +X_{33}\left[-\frac{2}{R}\frac{\partial^{2}w}{\partial x\partial \theta }\right.-\frac{1}{R^{2}}\left(\frac{1}{2}\psi +\varphi \right)\left.\frac{\partial u}{\partial \theta }+\frac{1}{R}\left(\frac{3}{2}\psi +\varphi \right)\frac{\partial v}{\partial x}\right]\\ +\varphi \left[\frac{X_{31}}{R}\frac{\partial u}{\partial x}+\frac{X_{32}}{R^{2}}\left(\frac{\partial v}{\partial \theta }+w\right)+\frac{X_{33}}{R}\left(\frac{\partial v}{\partial x}+\frac{1}{R}\frac{\partial u}{\partial \theta }\right)\right] \end{array}$$

where $X_{ij}=Y_{ij}h^{2}/12$, with i,j=1,2,3.

From Equations (11)–(16) it is noted that the integrating functions of the circumferential force $N_{\theta }$ and moment $M_{\theta }$ resultants are the same for the three different shell theories. Conversely, the integrating functions of the other force and moment resultants are different, since in the Flügge shell theory, they also include the term (1+z/R) (in this theory the thinness assumption is delayed), whereas this term is ignored in the other two thin shell theories. On the one hand, considering this additional term within the integrating functions of the resultants certainly makes the Flügge shell theory more refined (in fact, it is able to correctly model the vibrations of even relatively thick shells). On the other hand, this considerably increases the number of terms within the expressions of such resultants, and therefore, it strongly increases the computational effort of the numerical analyses. The main goal of this paper is therefore to verify whether a less refined theory, but with lower computational effort, such as the Donnell or Sanders shell theory, can provide sufficiently accurate results in terms of SWCNT natural frequencies compared to those provided by the Flügge shell theory, as investigated in [26] for cylindrical shells.

By substituting the expressions of the force and moment resultants (11)–(16) into the dynamic Equation (10), the equations of motion for the anisotropic elastic shell model are obtained in the form:

(17) $$\begin{array}{c} \left\{Y_{11}\frac{\partial^{2}}{{\partial x}^{2}}+\left(\frac{2Y_{13}}{R}-\varphi \frac{{3X}_{13}}{2R^{3}}\right)\frac{\partial^{2}}{\partial x\partial \theta }+\left[\frac{Y_{33}}{R^{2}}+\left(\psi -4\varphi \right)\frac{X_{33}}{4R^{4}}\right]\frac{\partial^{2}}{{\partial \theta }^{2}}\right\}u\\ +\left\{\left(Y_{13}+\varphi \frac{X_{13}}{R^{2}}\right)\frac{\partial^{2}}{{\partial x}^{2}}+\left(\frac{Y_{12}+Y_{33}}{R}-\psi \frac{3X_{33}}{4R^{3}}\right)\frac{\partial^{2}}{\partial x\partial \theta }\right.+\left[\frac{Y_{23}}{R^{2}}\right.\\ \left.\left.-\left(\psi +\varphi \right)\frac{X_{23}}{2R^{4}}\right]\frac{\partial^{2}}{{\partial \theta }^{2}}\right\}v+\left\{\left(\frac{Y_{12}}{R}-\varphi \frac{X_{12}}{R^{3}}\right)\frac{\partial }{\partial x}+\left(\frac{Y_{23}}{R^{2}}-\varphi \frac{X_{23}}{R^{4}}\right)\frac{\partial }{\partial \theta }\right.\\ -\varphi \frac{X_{11}}{R}\frac{\partial^{3}}{{\partial x}^{3}}+\left(1-6\varphi \right)\frac{X_{13}}{{2R}^{2}}\frac{\partial^{3}}{{\partial x}^{2}\partial \theta }+\left[\left(1-2\varphi \right)\frac{X_{33}}{R^{3}}-\varphi \frac{X_{12}}{R^{3}}\right]\\ \left.\frac{\partial^{3}}{\partial x{\partial \theta }^{2}}+\left(1-2\varphi \right)\frac{X_{23}}{2R^{4}}\frac{\partial^{3}}{{\partial \theta }^{3}}\right\}w=\rho h\frac{\partial^{2}u}{{\partial t}^{2}} \end{array}$$

(18) $$\begin{array}{c} \left\{\left(Y_{13}+\varphi \frac{3X_{13}}{2R^{2}}\right)\frac{\partial^{2}}{{\partial x}^{2}}+\left[\frac{Y_{12}+Y_{33}}{R}-\left(3\psi +4\varphi \right)\frac{X_{33}}{4R^{3}}\right]\frac{\partial^{2}}{\partial x\partial \theta }+\left[\frac{Y_{23}}{R^{2}}-\frac{X_{23}}{2R^{4}}\left(\psi +2\varphi \right)\right]\right.\\ \left.\frac{\partial^{2}}{{\partial \theta }^{2}}\right\}u+\left\{\left[Y_{33}+\left(9\psi +16\varphi \right)\frac{X_{33}}{{4R}^{2}}\right]\frac{\partial^{2}}{{\partial x}^{2}}+\left[\frac{2Y_{23}}{R}+\left(6\psi +5\varphi \right)\frac{X_{23}}{2R^{3}}\right]\right.\frac{\partial^{2}}{\partial x\partial \theta }+\left(\frac{Y_{22}}{R^{2}}\right.\\ \left.\left.+\psi \frac{X_{22}}{R^{4}}\right)\frac{\partial^{2}}{{\partial \theta }^{2}}\right\}v+\left\{\left(\frac{Y_{23}}{R}-\varphi \frac{X_{23}}{R^{3}}\right)\frac{\partial }{\partial x}+\left(\frac{Y_{22}}{R^{2}}-\varphi \frac{X_{22}}{R^{4}}\right)\right.\frac{\partial }{\partial \theta }-(3+2\varphi )\frac{X_{13}}{2R}\frac{\partial^{3}}{{\partial x}^{3}}\\ \left.-\left[\frac{X_{12}}{R^{2}}+\left(3+2\varphi \right)\frac{X_{33}}{R^{2}}\right]\frac{\partial^{3}}{{\partial x}^{2}\partial \theta }-(7+2\varphi )\frac{X_{23}}{2R^{3}}\frac{\partial^{3}}{\partial x{\partial \theta }^{2}}-\frac{X_{22}}{R^{4}}\frac{\partial^{3}}{{\partial \theta }^{3}}\right\}w=\rho h\frac{\partial^{2}v}{{\partial t}^{2}} \end{array}$$

(19) $$\begin{array}{c} \left[-\frac{Y_{12}}{R}\frac{\partial }{\partial x}-\frac{Y_{23}}{R^{2}}\frac{\partial }{\partial \theta }+\varphi \frac{X_{11}}{R}\frac{\partial^{3}}{{\partial x}^{3}}+\left(-\psi +4\varphi \right)\frac{X_{13}}{{2R}^{2}}\frac{\partial^{3}}{{\partial x}^{2}\partial \theta }-\psi \frac{X_{33}}{R^{3}}\frac{\partial^{3}}{\partial x{\partial \theta }^{2}}\right.\\ \left.-\left(\psi +2\varphi \right)\frac{X_{23}}{2R^{4}}\frac{\partial^{3}}{{\partial \theta }^{3}}\right]u+\left\{-\frac{Y_{23}}{R}\frac{\partial }{\partial x}-\frac{Y_{22}}{R^{2}}\frac{\partial }{\partial \theta }+\left(3\psi +4\varphi \right)\frac{X_{13}}{2R}\right.\frac{\partial^{3}}{{\partial x}^{3}}\\ +\left[\left(\psi +\varphi \right)\frac{X_{12}}{R^{2}}+\left(3\psi +4\varphi \right)\frac{X_{33}}{R^{2}}\right]\frac{\partial^{3}}{{\partial x}^{2}\partial \theta }+\left(7\psi +6\varphi \right)\frac{X_{23}}{2R^{3}}\frac{\partial^{3}}{\partial x{\partial \theta }^{2}}\left.+\psi \frac{X_{22}}{R^{4}}\frac{\partial^{3}}{{\partial \theta }^{3}}\right\}v\\ +\left[-\frac{Y_{22}}{R^{2}}-\varphi \frac{X_{22}}{R^{4}}\frac{\partial^{2}}{{\partial \theta }^{2}}-X_{11}\frac{\partial^{4}}{{\partial x}^{4}}-\frac{4X_{13}}{R}\frac{\partial^{4}}{{\partial x}^{3}\partial \theta }-\left(\frac{{2X}_{12}+{4X}_{33}}{R^{2}}\right)\right.\\ \left.\frac{\partial^{4}}{{\partial x}^{2}{\partial \theta }^{2}}-\frac{4X_{23}}{R^{3}}\frac{\partial^{4}}{\partial x{\partial \theta }^{3}}-\frac{X_{22}}{R^{4}}\frac{\partial^{4}}{{\partial \theta }^{4}}\right]w=\rho h\frac{\partial^{2}w}{{\partial t}^{2}} \end{array}$$

## 5. Solution Method

In this paper, a complex method is considered to analytically solve the dynamic equations of motions (17)–(19) and to obtain the natural frequencies of SWCNTs. Specifically, a complex variable is used to solve the partial differential Equations (17)–(19) by setting the real and imaginary zero.

In the present work, simply supported boundary conditions are adopted. These boundary conditions, for the complex method, impose the conditions $Re\left(v\right)=Re\left(w\right)=0$ at both ends x=(0,L) of the SWCNT. The displacement field that satisfies these boundary conditions can be written as [39]:

(20) $$\begin{array}{c} u(x,\theta ,t)=\overset{-}{U}\exp\left(i\lambda_{q}x\right)\cos\left(s\theta \right)\cos\left(\omega t\right)\\ v(x,\theta ,t)=-i\overset{-}{V}\exp\left(i\lambda_{q}x\right)\sin\left(s\theta \right)\cos\left(\omega t\right)\\ w(x,\theta ,t)=-i\overset{-}{W}\exp\left(i\lambda_{q}x\right)\cos\left(s\theta \right)\cos\left(\omega t\right) \end{array}$$

where $\left(\overset{-}{U},\overset{-}{V},\overset{-}{W}\right)$ denote the displacement amplitudes along the longitudinal u, circumferential v, and radial w directions, respectively; i is the imaginary unit; $\lambda_{q}$ is the wavenumber along the longitudinal direction, with $\lambda_{q}=q\pi /L$, where q is the number of longitudinal half-waves and L is the length of the SWCNT; s is the number of circumferential waves; and ω is the circular frequency.

Substituting Equation (20) into Equations (17)–(19), a set of algebraic equations for the displacement amplitudes $\left(\overset{-}{U},\overset{-}{V},\overset{-}{W}\right)$ is obtained, which can be written in the form [39]:

(21) $$E{\left(\lambda_{q},s,\omega \right)}_{3\times 3}\left[\begin{array}{c} \overset{-}{U}\\ \overset{-}{V}\\ \overset{-}{W} \end{array}\right]=\left[\begin{array}{c} 0\\ 0\\ 0 \end{array}\right]$$

where E is a non-symmetric matrix, whose elements are:

(22) $$E_{11}=Y_{11}\lambda_{q}^{2}+\left(\frac{2Y_{13}}{R}-\varphi \frac{{3X}_{13}}{2R^{3}}\right)\lambda_{q}s+\left[\frac{Y_{33}}{R^{2}}+\left(\psi -4\varphi \right)\frac{X_{33}}{4R^{4}}\right]s^{2}-\rho h\omega^{2}$$

(23) $$E_{12}=-\left(Y_{13}+\varphi \frac{X_{13}}{R^{2}}\right)\lambda_{q}^{2}-\left(\frac{Y_{12}+Y_{33}}{R}\right.\left.-\psi \frac{3X_{33}}{4R^{3}}\right)\lambda_{q}s-\left[\frac{Y_{23}}{R^{2}}-\left(\psi +\varphi \right)\frac{X_{23}}{2R^{4}}\right]s^{2}$$

(24) $$\begin{array}{c} E_{13}=-\left(\frac{Y_{12}}{R}-\varphi \frac{X_{12}}{R^{3}}\right)\lambda_{q}-\left(\frac{Y_{23}}{R^{2}}-\varphi \frac{X_{23}}{R^{4}}\right)s-\varphi \frac{X_{11}}{R}\lambda_{q}^{3}\\ +(1-6\varphi )\frac{X_{13}}{2R^{2}}\lambda_{q}^{2}s+\left[(1-2\varphi )\frac{X_{33}}{R^{3}}-\varphi \frac{X_{12}}{R^{3}}\right]\lambda_{q}s^{2}+\left(1-2\varphi \right)\frac{X_{23}}{2R^{4}}s^{3} \end{array}$$

(25) $$E_{21}=-\left(Y_{13}+\varphi \frac{3X_{13}}{2R^{2}}\right)\lambda_{q}^{2}-\left[\frac{Y_{12}+Y_{33}}{R}\right.\left.-\left(3\psi +4\varphi \right)\frac{X_{33}}{4R^{3}}\right]\lambda_{q}s-\left[\frac{Y_{23}}{R^{2}}-\left(\psi +2\varphi \right)\frac{X_{23}}{2R^{4}}\right]s^{2}$$

(26) $$E_{22}=\left[Y_{33}+\left(9\psi +16\varphi \right)\frac{X_{33}}{4R^{2}}\right]\lambda_{q}^{2}+\left[\frac{2Y_{23}}{R}\right.\left.+\left(6\psi +5\varphi \right)\frac{X_{23}}{{2R}^{3}}\right]\lambda_{q}s+\left(\frac{Y_{22}}{R^{2}}+\psi \frac{X_{22}}{R^{4}}\right)s^{2}-\rho h\omega^{2}$$

(27) $$\begin{array}{c} E_{23}=\left(\frac{Y_{23}}{R}-\varphi \frac{X_{23}}{R^{3}}\right)\lambda_{q}+\left(\frac{Y_{22}}{R^{2}}-\varphi \frac{X_{22}}{R^{4}}\right)s+\left(3+2\varphi \right)\frac{X_{13}}{2R}\lambda_{q}^{3}\\ +\left[\frac{X_{12}}{R^{2}}+(3+2\varphi )\frac{X_{33}}{R^{2}}\right]\lambda_{q}^{2}s+\left(7+2\varphi \right)\frac{X_{23}}{2R^{3}}\lambda_{q}s^{2}+\frac{X_{22}}{R^{4}}s^{3} \end{array}$$

(28) $$E_{31}=-\frac{Y_{12}}{R}\lambda_{q}+\frac{Y_{23}}{R^{2}}s-\varphi \frac{X_{11}}{R}\lambda_{q}^{3}-\left(\psi -4\varphi \right)\frac{X_{13}}{2R^{2}}\lambda_{q}^{2}s+\psi \frac{X_{33}}{R^{3}}\lambda_{q}s^{2}-\left(\psi +2\varphi \right)\frac{X_{23}}{2R^{4}}s^{3}$$

(29) $$\begin{array}{c} E_{32}=-\frac{Y_{23}}{R}\lambda_{q}+\frac{Y_{22}}{R^{2}}s-\left(3\psi +4\varphi \right)\frac{X_{13}}{2R}\lambda_{q}^{3}+\left[(\psi +\varphi )\frac{X_{12}}{R^{2}}\right.\\ \left.+(3\psi +4\varphi )\frac{X_{33}}{R^{2}}\right]\lambda_{q}^{2}s-(7\psi +6\varphi )\frac{X_{23}}{2R^{3}}\lambda_{q}s^{2}+\psi \frac{X_{22}}{R^{4}}s^{3} \end{array}$$

(30) $$E_{33}=\frac{Y_{22}}{R^{2}}+X_{11}\lambda_{q}^{4}-\varphi \frac{X_{22}}{R^{4}}s^{2}-\frac{4X_{13}}{R}\lambda_{q}^{3}s+\left(\frac{2X_{12}+4X_{33}}{R^{2}}\right)\lambda_{q}^{2}s^{2}-\frac{4X_{23}}{R^{3}}\lambda_{q}s^{3}+\frac{X_{22}}{R^{4}}s^{4}-\rho h\omega^{2}$$

At this point it is useful to remember that by imposing the parameters (ψ=0,φ=0), we obtain the elements of matrix E for the Donnell shell theory; by imposing the parameters (ψ=1,φ=0), we obtain the elements of matrix E for the Sanders shell theory; and by imposing the parameters (ψ=0,φ=1), we obtain the elements of matrix E for the Flügge shell theory. See Expansion (2) for the middle surface change in the curvature $k_{\theta }$ and torsion $k_{x\theta }$ of the shell.

For the non-trivial solution, the determinant of the set of Equation (21) must be equal to zero [40]:

(31) $$\det{E(\lambda_{q},s,\omega )}_{3\times 3}=0$$

Solving Equation (31), we get a third-order algebraic equation in $\omega^{2}$; this last equation provides three different eigenfrequencies for each number of waves (q,s) that give three different vibration modes (i.e., longitudinal, torsional and radial modes). Since the highest natural frequency corresponds to the radial vibration mode, only the radial natural frequencies were computed in the numeric results.

## 6. Numeric Results

In this paper, the natural frequencies of SWCNTs were obtained in the framework of the Donnell, Sanders, and Flügge shell theories. An anisotropic elastic shell model was used to take into account the chirality effects of CNTs. Simply supported boundary conditions were imposed. Vibration modes with different number of waves along the longitudinal and circumferential directions were considered. SWCNTs with different values of radius R and aspect ratio L/R were investigated.

As known from the literature, two relevant open issues related to the continuous modelling of carbon nanotubes are due to their intrinsic anisotropic character and their discrete configuration. To this end, it is very important to adopt parameters and models able to correctly describe the actual molecular structure of carbon nanotubes.

In Table 1, the values of carbon-carbon bond parameters $\left(a,k_{\rho },k_{\theta }\right)$ and equivalent continuous parameters (h,ρ) retrieved from the literature are reported. In particular, parameters $k_{\rho }$ and $k_{\theta }$, which denote force constants correlated to the variance of carbon-carbon bond length a and angle θ, respectively, were adopted to express the anisotropic elastic constants of SWCNTs via the molecular mechanics-based “stick-spiral model” developed by Chang [35].

Moreover, in order to study the dynamics of the actual discrete CNT via a continuous cylindrical shell, an equivalent thickness h, which is derived from MD simulations of CNT energy, and an equivalent mass density ρ, resulting from graphite surface density, were considered; see Ref. [16] for more details.

### 6.1. Comparison of the Shell Theories with the Results of Molecular Dynamics Simulations

In this section, the natural frequencies of the radial breathing mode (q=0,s=0) of the SWCNT of Table 1 with an aspect ratio L/R=10 obtained by considering Donnell, Sanders, and Flügge shell theories are compared with the results of molecular dynamics simulations available in literature for different chirality indices (n,m); see Table 2.

Specifically, the results of the molecular dynamics simulations reported in Table 2 were retrieved from Ref. [10]. In that work, the free vibrations of armchair, zigzag, and chiral SWCNTs with different aspect ratios and diameters were studied via MM3 potential. This potential considers bond stretching, change in angles between adjacent bonds, torsion of the bond, van der Waals forces, and the coupling among stretching, bending, and torsional deformations. In particular, the energy due to the bond stretching has terms that are quadratic, cubic, and quartic in the bond length; thus, the strain energy due to the bond stretching is not an even function of the change in the bond length; see Ref. [10] for more details.

Obviously, the results of molecular dynamics simulations are the most correct since these simulations are able to correctly take into account the actual molecular structure of carbon nanotubes. However, the computational effort of molecular dynamics simulations is very high, in particular when dealing with very long and thin structures (such as carbon nanotubes), which have an extremely large number of atoms, and therefore, these results are available only for a reduced range of CNT geometries. To this end, it seems useful to investigate which shell theory is more accurate in the vibration modelling of carbon nanotubes considered as continuous homogeneous structures.

From Table 2, it can be observed that for all considered chirality indices, by assuming molecular dynamics results as reference, the percentage differences of the Flügge shell theory are the lowest, the percentage differences of the Donnell shell theory are the highest, and the Sanders shell theory gives an intermediate response.

These results could easily be predicted considering the strain–displacement Relationship (2), in which the Donnell shell theory has fewer terms than the other two (and therefore is the least accurate), and observing the force and moment Resultants (11)–(16), in which the Flügge shell theory includes more terms (and therefore is the most accurate).

Even if, among the three considered shell theories, Flügge is obtained to be the most accurate, it is preferable to not adopt this theory, because it has a relatively high computational effort, due to the large number of terms in the expansions of the force and moment resultants. This aspect is relevant not so much in the study of the linear vibrations (natural frequencies) but instead in the analysis of the non-linear vibrations (amplitude-frequency responses), where in the strain–displacement relationships the non-linear terms are also considered, and therefore the computational weight in solving the corresponding dynamic equations of motion becomes much greater.

To this aim, it could be useful to analyse whether the Donnell or Sanders shell theories are sufficiently accurate in the continuous modelling of SWCNT vibrations. Therefore, in the following, the natural frequencies of simply supported SWCNTs with different geometries and wavenumbers will be computed in the framework of the Donnell and Sanders shell theories, where the results of Flügge shell theory will be considered as reference.

### 6.2. Comparison of the Shell Theories for Different SWCNT Geometries and Wavenumbers

In the present section, the natural frequencies of the simply supported SWCNT of Table 1 obtained by considering the Donnell, Sanders, and Flügge shell theories were compared for different chirality indices (n,m), aspect ratios L/R, numbers of longitudinal half-waves q, and circumferential waves s.

By taking as reference the results of the Flügge shell theory (which are the closest to those obtained from molecular dynamics simulations, see Table 2), we attempted to investigate the fields of applicability and limitations of the Donnell and Sanders shell theories.

**Figure 2:**
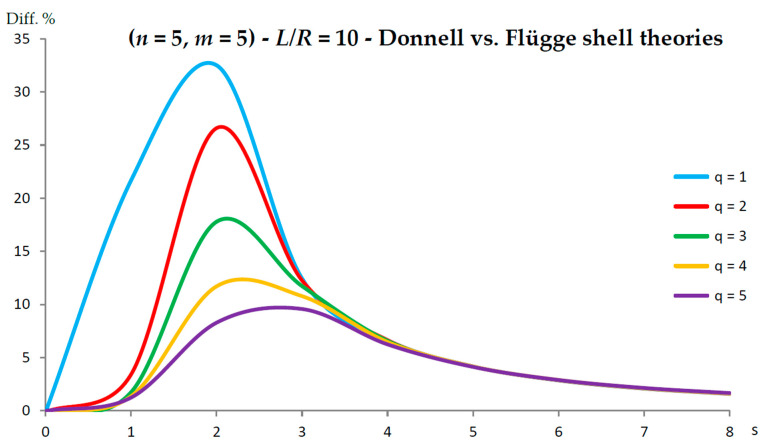
Percentage differences between the radial natural frequencies obtained via Donnell and Flügge shell theories (Flügge as the reference). Anisotropic elastic shell model. Simply supported SWCNT of Table 1 with chirality indices (n=5,m=5) and aspect ratio L/R=10. Number of longitudinal half-waves q. Number of circumferential waves s.

**Figure 3:**
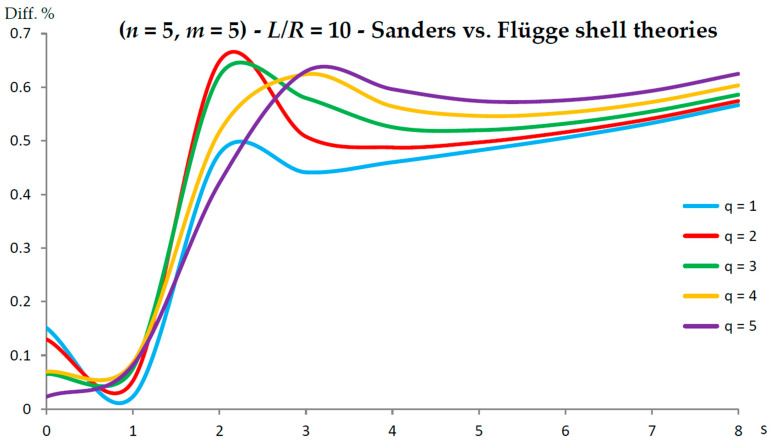
Percentage differences between the radial natural frequencies obtained via Sanders and Flügge shell theories (Flügge as the reference). Anisotropic elastic shell model. Simply supported SWCNT of Table 1 with chirality indices (n=5,m=5) and aspect ratio L/R=10. Number of longitudinal half-waves q. Number of circumferential waves s.

**Figure 4:**
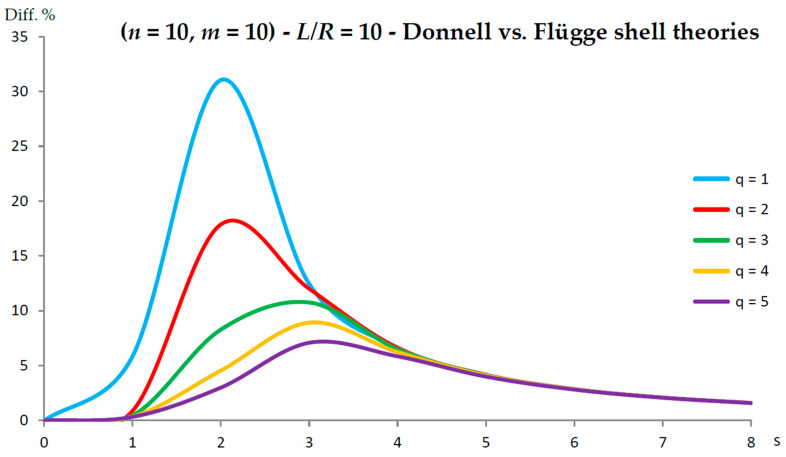
Percentage differences between the radial natural frequencies obtained via Donnell and Flügge shell theories (Flügge as the reference). Anisotropic elastic shell model. Simply supported SWCNT of Table 1 with chirality indices (n=10,m=10) and aspect ratio L/R=10. Number of longitudinal half-waves q. Number of circumferential waves s.

**Figure 5:**
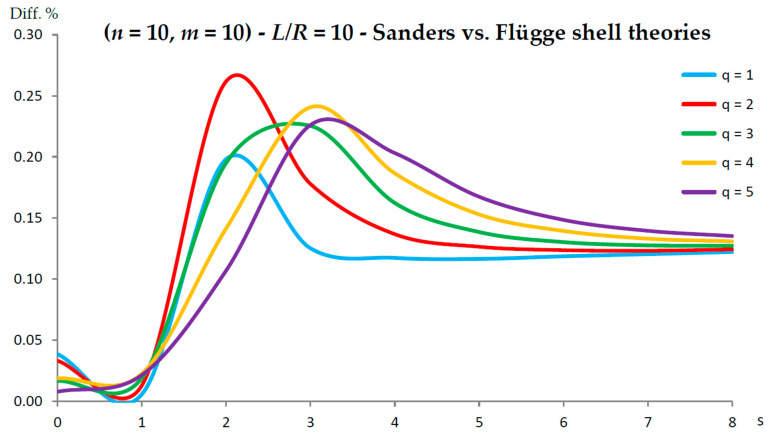
Percentage differences between the radial natural frequencies obtained via Sanders and Flügge shell theories (Flügge as the reference). Anisotropic elastic shell model. Simply supported SWCNT of Table 1 with chirality indices (n=10,m=10) and aspect ratio L/R=10. Number of longitudinal half-waves q. Number of circumferential waves s.

**Figure 6:**
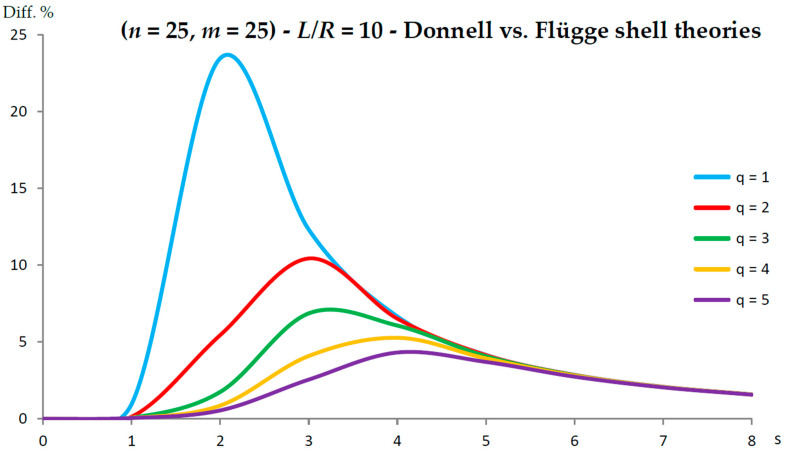
Percentage differences between the radial natural frequencies obtained via Donnell and Flügge shell theories (Flügge as the reference). Anisotropic elastic shell model. Simply supported SWCNT of Table 1 with chirality indices (n=25,m=25) and aspect ratio L/R=10. Number of longitudinal half-waves q. Number of circumferential waves s.

**Figure 7:**
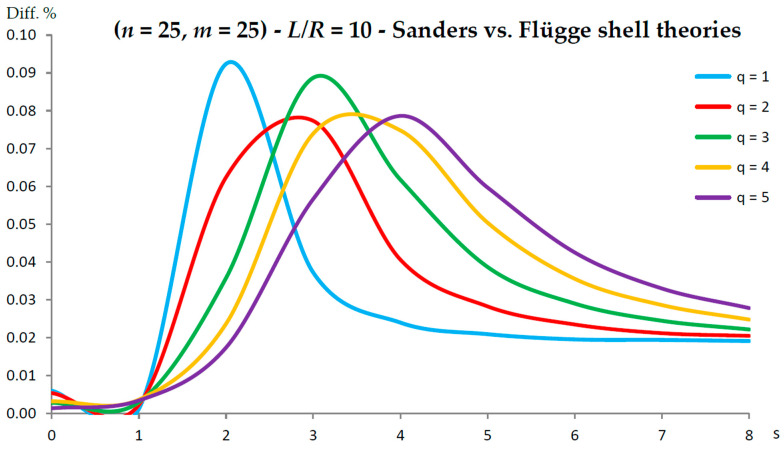
Percentage differences between the radial natural frequencies obtained via Sanders and Flügge shell theories (Flügge as the reference). Anisotropic elastic shell model. Simply supported SWCNT of Table 1 with chirality indices (n=25,m=25) and aspect ratio L/R=10. Number of longitudinal half-waves q. Number of circumferential waves s.

**Figure 8:**
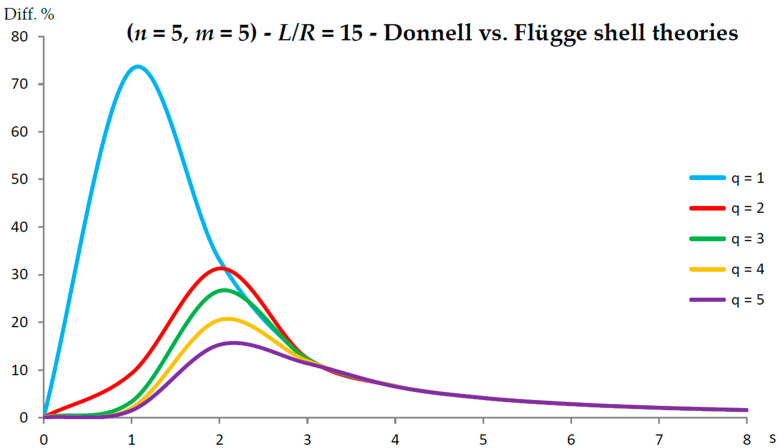
Percentage differences between the radial natural frequencies obtained via Donnell and Flügge shell theories (Flügge as the reference). Anisotropic elastic shell model. Simply supported SWCNT of Table 1 with chirality indices (n=5,m=5) and aspect ratio L/R=15. Number of longitudinal half-waves q. Number of circumferential waves s.

**Figure 9:**
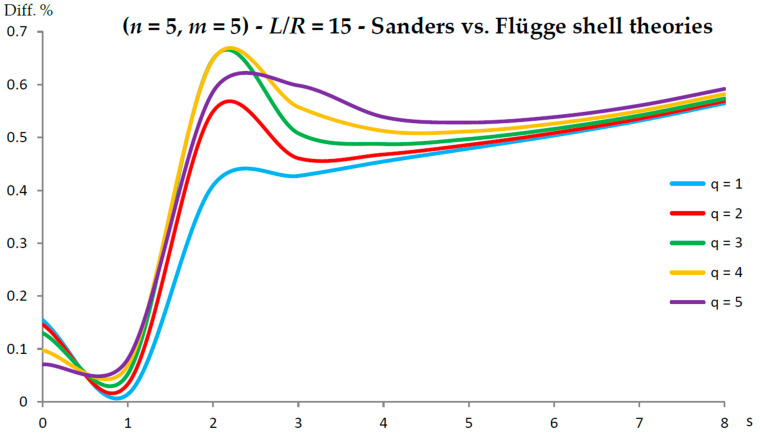
Percentage differences between the radial natural frequencies obtained via Sanders and Flügge shell theories (Flügge as the reference). Anisotropic elastic shell model. Simply supported SWCNT of Table 1 with chirality indices (n=5,m=5) and aspect ratio L/R=15. Number of longitudinal half-waves q. Number of circumferential waves s.

**Figure 10:**
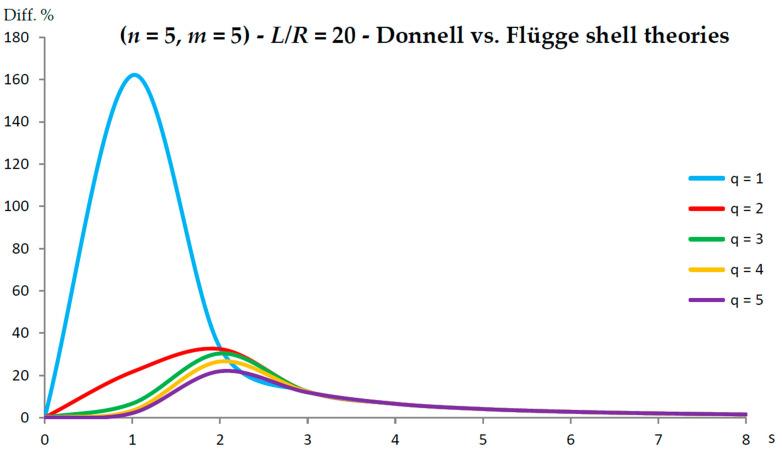
Percentage differences between the radial natural frequencies obtained via Donnell and Flügge shell theories (Flügge as the reference). Anisotropic elastic shell model. Simply supported SWCNT of Table 1 with chirality indices (n=5,m=5) and aspect ratio L/R=20. Number of longitudinal half-waves q. Number of circumferential waves s.

**Figure 11:**
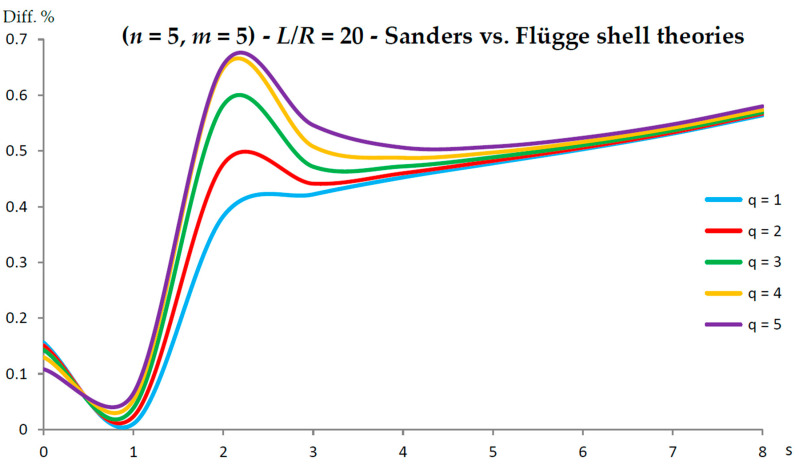
Percentage differences between the radial natural frequencies obtained via Sanders and Flügge shell theories (Flügge as the reference). Anisotropic elastic shell model. Simply supported SWCNT of Table 1 with chirality indices (n=5,m=5) and aspect ratio L/R=20. Number of longitudinal half-waves q. Number of circumferential waves s.

Figure 2, Figure 3, Figure 4, Figure 5, Figure 6, Figure 7, Figure 8, Figure 9, Figure 10 and Figure 11 show the comparisons between Donnell and Flügge shell theories, and between Sanders and Flügge shell theories, carried out by adopting increasing values of chirality indices (SWCNT radius), specifically (n=5,m=5) (i.e., R=0.34 nm), (n=10,m=10) (i.e., R=0.68 nm), and (n=25,m=25) (i.e., R=1.70 nm), and increasing values of SWCNT aspect ratio L/R=(10,15,20) are shown.

It must be underlined that this analysis was carried out within the range commonly assumed for the radius of CNTs. In fact, it is reported that, “a single-wall nanotube is defined by a cylindrical graphene sheet with a diameter of about 0.5–10.0 nm, though most of the observed single-wall nanotubes have diameters < 2 nm”, see Ref. [9].

As for the Donnell shell theory, when increasing the radius R, the percentage difference with respect to the Flügge shell theory decreased for all longitudinal wavenumbers q, where the maximum value is obtained at q=1 and decreased with increasing q; the maximum peak corresponding to q=1 was always located at s=2, whereas the peaks corresponding to q=(2−5) moved to higher circumferential wavenumbers.

On the other hand, when increasing the aspect ratio L/R, the percentage difference with respect to Flügge shell theory increased for all longitudinal wavenumbers q, where the maximum value was found again at q=1 and decreased with increasing q, but now the maximum peak corresponding to q=1 moved from s=2 to s=1 (lower circumferential wavenumber), whereas the peaks corresponding to q=(2−5) were always located at s=2.

It should be underlined that the increase in the percentage difference at the longitudinal wavenumber q=1 obtained by increasing the aspect ratio L/R is extremely higher than the corresponding decrease at the longitudinal wavenumber q=1 obtained by increasing the radius R (i.e., the effect of aspect ratio is prevalent with respect to radius).

As for Sanders shell theory, increasing both the radius R and the aspect ratio L/R, it was always found that the percentage difference with respect to the Flügge shell theory was relatively low for every number of longitudinal and circumferential waves (<1%).

In particular, when increasing the radius R, the maximum percentage difference moved from q=2 to q=1, decreasing its value (from 0.7% to 0.1%); differently, when increasing the aspect ratio L/R, the maximum percentage difference moved from q=2 to q=5, preserving its value (0.7%) (i.e., no effect of both aspect ratio and radius).

Therefore, by considering the parametric analyses presented in Figure 2, Figure 3, Figure 4, Figure 5, Figure 6, Figure 7, Figure 8, Figure 9, Figure 10 and Figure 11, it can be observed that the Donnell shell theory cannot be applied for the vibration modelling of SWCNTs with relatively low radius R and relatively high aspect ratio L/R, and for the vibration modelling of modes with relatively low numbers of longitudinal q and circumferential s waves. However, as previously reported, the effect of the aspect ratio is prevalent with respect to the radius (and also to the wavenumbers) in providing the very high percentage difference obtained between the Donnell and Flügge shell theories. This is due to the different expression of the middle surface change in curvature $k_{\theta }$ and torsion $k_{x\theta }$ of the shell for the two theories (see the strain–displacement Equation (2)), which present more terms in the Flügge than in the Donnell shell theory, and in particular to the middle surface torsion, which is very sensitive to the value of aspect ratio.

Specifically, for the relatively low value of aspect ratio L/R=10, the maximum peak of percentage difference was located at the circumferential flexure mode (q=1,s=2), where the effect of the middle surface change in the curvature $k_{\theta }$ was prevalent; see Figure 2. On the other hand, for the relatively high value of aspect ratio L/R=20, the maximum peak of percentage difference was located at the beam-like mode (q=1,s=1), where the effect of the middle surface torsion $k_{x\theta }$ prevailed; see Figure 10.

Conversely, by considering the parametric analyses presented in Figure 2, Figure 3, Figure 4, Figure 5, Figure 6, Figure 7, Figure 8, Figure 9, Figure 10 and Figure 11, it can be observed that Sanders shell theory is able to model with very good accuracy the linear vibrations of SWCNTs for all considered geometries and wavenumbers, and therefore it can be adopted instead of the more complex Flügge shell theory to compute the natural frequencies of SWCNTs. This is due to the fact that, in the Sanders shell theory, the effect of the aspect ratio is not present (as the strain–displacement Equation (2) is very similar to that of the Flügge shell theory), but only the effect of the radius occurs.

In fact, from the numerical simulations carried out in the present work, it was found that for both the Donnell and Sanders shell theories, the difference in the natural frequencies with respect to the Flügge shell theory decreased with CNT radius, see as e.g., Figure 2 compared to Figure 6 (Donnell vs. Flügge), and Figure 3 compared to Figure 7 (Sanders vs. Flügge). This behaviour can be understood by observing once more the expressions of the middle surface change in curvature $k_{\theta }$ and torsion $k_{x\theta }$ of the shell; see Equation (2). Since in these two expressions, the radius R is located in the denominator, then, by increasing the radius R, their value (i.e., their influence) reduces, and therefore, the natural frequencies of the three theories tend to become closer.

**Figure 12:**
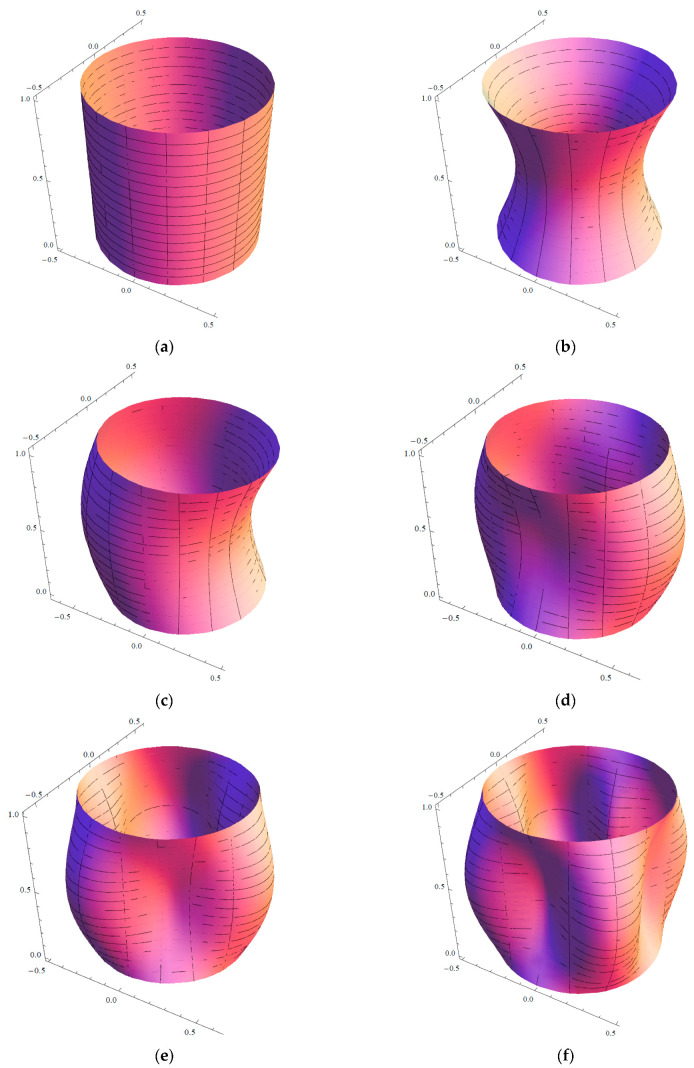
Mode shapes of the simply supported SWNT of Table 1 with chirality indices (n=5,m=5) and aspect ratio L/R=10: (**a**) Radial breathing mode (q=0,s=0); (**b**) axisymmetric mode (q=1,s=0); (**c**) beam-like mode (q=1,s=1); (**d**) shell-like mode (q=1,s=2); (**e**) shell-like mode (q=1,s=3); and (**f**) shell-like mode (q=1,s=4).

Finally, in Figure 12, six different mode shapes of the simply supported SWCNT of Table 1 with chirality indices (n=5,m=5) and aspect ratio L/R=10 are shown, where the radial breathing mode (q=0,s=0) corresponds to Rayleigh’s inextensional symmetrical mode (i.e., uniform vibration). Moreover, it also shows the axisymmetric mode (q=1,s=0) (no circumferential waves), beam-like mode (q=1,s=1) (one circumferential wave, characteristic of structures with a very long aspect ratio, such as beams), and shell-like modes (q=1,s=2−4) (two or more circumferential waves, characteristic of three-dimensional thin-walled structures, such as circular cylindrical shells). Such a graphical representation of the vibration modes could be useful for interpreting the previous results and comparisons.

## 7. Conclusions

In this paper, the natural frequencies of SWCNTs obtained in the framework of the Donnell, Sanders, and Flügge shell theories were compared. An anisotropic elastic shell model was adopted to take into account the intrinsic chirality effects of CNTs. Simply supported boundary conditions were imposed. Vibration modes with different wavenumbers along the longitudinal and circumferential directions were studied. SWCNTs with different values of radius R and aspect ratio L/R were considered. The most important findings of the present paper are reported below.

- By means of comparisons with the results of molecular dynamic simulations reported in the literature, it was derived that the Flügge shell theory is the most accurate in the computation of the natural frequencies of SWCNTs.
- Since the Flügge shell theory requires a very high computational effort due to the large number of terms in the equations of motion, it was investigated whether a simpler shell theory is able to model with sufficient accuracy the linear vibrations of SWCNTs.
- It was found that the Donnell shell theory was not accurate for relatively low longitudinal and circumferential wavenumbers, for relatively low diameters, and for relatively high aspect ratios, and therefore, it is not able to properly model SWCNT vibrations.
- On the other hand, it was found that the Sanders shell theory was very accurate for all considered geometries and wavenumbers, and therefore, it can be adopted instead of the more complex Flügge shell theory to properly model SWCNT vibrations.

As first application of the results obtained in the present paper, the authors are planning to write a new manuscript on the effects of nonlocal elasticity and strain gradient on the linear vibrations of SWCNTs by considering an anisotropic elastic model in the framework of the Sanders shell theory.

A second relevant application of the findings of the present work regards the nonlinear vibrations of CNTs. It has been proven in the literature that, in the presence of a large number of carbon atoms, i.e., for relatively long or multi-walled carbon nanotubes, molecular dynamics simulations require higher computational effort than all equivalent continuous models, including the Flügge shell theory [3]. Moreover, by carrying out numerical simulations in linear field, it was found that all equivalent continuous models, among which are the Donnell, Sanders, and Flügge shell theories, are equally time costing [23]. The problem with the Flügge shell theory is due to the transition from linear to nonlinear analysis. On the one hand, the additional terms present in the expressions of forces and moments give the Flügge shell theory greater accuracy than that of Sanders and Donnell. On the other hand, these terms lead to very high computational effort in the numerical simulations in the nonlinear field, which are necessary to study the actual dynamic behaviour of carbon nanotubes and to investigate fluid–structure interactions. For this reason, especially for the nonlinear analyses, it is preferable to use the Sanders shell theory, since it is more accurate than the Donnell one (see the results of the present paper) and less computationally expensive than the Flügge one.

## Figures and Tables

**Table 1:** Mechanical parameters adopted in the anisotropic elastic continuous shell model [16,35].

C-C bond length a (nm)	0.142
C-C bond elongation $k_{\rho }$ (nN/nm)	742
C-C bond angle variance $k_{\theta }$ (nN·nm)	1.42
Shell thickness h (nm)	0.0665
Mass density ρ (kg/m^3^)	11,700

**Table 2:** Natural frequencies of the radial breathing mode (q=0,s=0) of the SWCNT of Table 1 with aspect ratio. L/R=10. Comparisons among Donnell, Sanders, Flügge shell theories and molecular dynamics simulations.

$\mathrm{Natural}\;\mathrm{Frequency}\;\omega_{RBM}$ (cm^−1^)		Difference (%)		
Chirality Indices (n,m)	Molecular Dynamics Simulations [10]	Donnell Shell Theory	Sanders Shell Theory	Flügge Shell Theory
(10, 0)	290.463	2.38	1.32	0.42
(9, 6)	221.496	3.47	1.93	0.43
(8, 8)	209.008	4.05	2.25	0.51
(9, 9)	185.896	4.03	2.24	0.49
(16, 0)	181.747	3.10	1.72	0.04
(10, 10)	167.377	4.01	2.23	0.48
(11, 11)	152.207	4.00	2.22	0.47
(20, 0)	145.363	3.35	1.86	0.11
(22, 7)	110.808	3.71	2.06	0.32
(25, 10)	93.153	3.56	1.98	0.21
(30, 5)	88.750	3.51	1.95	0.16
(36, 5)	75.118	3.47	1.93	0.17
(33, 16)	67.221	3.56	1.98	0.23

## Data Availability

All data are available from the authors.
